# The Atherosclerotic Profile of a Young Symptomatic Population between 19 and 49 Years: Coronary Computed Tomography Angiography or Coronary Artery Calcium Score?

**DOI:** 10.3390/jcdd8110157

**Published:** 2021-11-18

**Authors:** Gudrun Maria Feuchtner, Christoph Beyer, Christian Langer, Sven Bleckwenn, Thomas Senoner, Fabian Barbieri, Anna Luger, Philipp Spitaler, Gerlig Widmann, Agne Adukauskaite, Wolfgang Dichtl, Guy Friedrich, Fabian Plank

**Affiliations:** 1 Department of Radiology, Medical University of Innsbruck, 6020 Innsbruck, Austria; Christian.Langer@tirol-kliniken.at (C.L.); Sven.Bleckwenn@gmx.de (S.B.); Anna.Luger@i-med.ac.at (A.L.); Gerlig.Widmann@i-med.ac.at (G.W.); 2 Department of Internal Medicine III, Medical University of Innsbruck, 6020 Innsbruck, Austria; Christoph.Beyer@i-med.ac.at (C.B.); Thomas.Senoner@i-med.ac.at (T.S.); Fabian.Barbieri@i-med.ac.at (F.B.); Philipp.Spitaler@i-med.ac.at (P.S.); Agne.Adukauskaite@tirol-kliniken.at (A.A.); Wolfgang.Dichtl@i-med.ac.at (W.D.); Guy.Friedrich@tirol-kliniken.at (G.F.); Fabian.Plank@i-med.ac.at (F.P.); 3 Department of Cardiology, Charité-Universitätsmedizin Berlin, Corporate Member of Freie Universität Berlin, Humboldt-Universität zu Berlin, and Berlin Institute of Health, 12203 Berlin, Germany

**Keywords:** atherosclerosis, coronary arteries, imaging, computed tomography, young high-risk population

## Abstract

(1) Background: Whether coronary computed tomography angiography (CTA) or the coronary artery calcium score (CACS) should be used for diagnosis of coronary heart disease, is an open debate. The aim of our study was to compare the atherosclerotic profile by coronary CTA in a young symptomatic high-risk population (age, 19–49 years) in comparison with the coronary artery calcium score (CACS). (2) Methods: 1137 symptomatic high-risk patients between 19–49 years (mean age, 42.4 y) who underwent coronary CTA and CACS were stratified into six age groups. CTA-analysis included stenosis severity and high-risk-plaque criteria (3) Results: Atherosclerosis was more often detected based on CTA than based on CACS (45 vs. 27%; *p* < 0.001), 50% stenosis in 13.6% and high-risk plaque in 17.7%. Prevalence of atherosclerosis was low and not different between CACS and CTA in the youngest age groups (19–30 y: 5.2 and 6.4% and 30–35 y: 10.6 and 16%). In patients older than >35 years, the rate of atherosclerosis based on CTA increased (*p* = 0.004, OR: 2.8, 95%CI:1.45–5.89); and was higher by CTA as compared to CACS (34.9 vs. 16.7%; *p* < 0.001), with a superior performance of CTA. In patients older than 35 years, stenosis severity (*p* = 0.002) and >50% stenosis increased from 2.6 to 12.5% (*p* < 0.001). High-risk plaque prevalence increased from 6.4 to 26.5%. The distribution of high-risk plaque between CACS 0 and >0.1 AU was similar among all age groups, with an increasing proportion in CACS > 0.1 AU with age. A total of 24.9% of CACS 0 patients had coronary artery disease based on CTA, 4.4% > 50% stenosis and 11.5% had high-risk plaque. (4) Conclusions: In a symptomatic young high-risk population older than 35 years, CTA performed superior than CACS. In patients aged 19–35 years, the rate of atherosclerosis was similar and low based on both modalities. CACS 0 did not rule out coronary artery disease in a young high-risk population.

## 1. Introduction

The coronary artery calcium score (CACS) is a screening tool for coronary artery disease (CAD) in asymptomatic low-to-intermediate risk patients, based on trials mainly enrolling adults older than 50 years [1,2,3].

Coronary artery calcium has been also associated with a 3-up to 5-fold increased risk of fatal and nonfatal coronary heart disease events in younger individuals between 32 and 46 years of age [1], and CACS of >100 Agatston Units (AU) with early death [1]. The CACS has an excellent negative prognostic value, as shown in numerous large cohorts [3] consisting mainly of elderly adults, however its positive predictive value is lower [4]. Recent data showed that coronary heart disease mortality rate is also low for younger patients aged 30–49 years with CACS 0 [2]. However, mortality is naturally lower in this age group, while non-fatal STEMI or NSTEMI–ACS occurs more often, which were not included as study endpoints in most of those trials [2,4]. The majority of culprit lesions in acute coronary syndromes show signs of plaque vulnerability [5,6], such as a lipid-rich low-attenuation plaque (LAP < 30 HU) [6] and others based on coronary computed tomography angiography (CTA)(“high-risk-plaque” = HRP-criteria) [6,7,8,9,10]. Especially in younger individuals, early stages of atherosclerosis—without a calcified plaque component—may be more common. Accordingly, coronary CTA may be a superior imaging modality in this population, due to its ability to detect high-risk plaque and graduate stenosis severity [11]. High-risk plaque (HRP) is a novel imaging biomarker that predicts major adverse cardiac events, independent of cardiovascular risk factors, the CACS, and stenosis severity [6]. 

Coronary CTA is recommended by the ESC 2019 as Class I indication in patients with chronic coronary syndromes and low-to-intermediate and high pre-test probability [12]. The coronary artery disease burden based on CTA in relationship to CACS is not well described in young adults < 49 years of age. In the largest series of 914 asymptomatic healthy Koreans (<45 years), only 9.4% had subclinical atherosclerosis, and the rate of stenosis > 50% was very low with 0.1% [13]. No data exist for Caucasians, and no large cohorts of young “high-risk” symptomatic individuals have been investigated.

Therefore, our objective was to define the coronary artery disease profile by coronary CTA (stenosis severity and high-risk plaque criteria) in a symptomatic high-risk population of young adults aged 19–49 years in comparison with the coronary artery calcium score, stratified into six different age groups. 

## 2. Materials and Methods

We screened our hospital database for cardiac CTAs performed between 2005 and 06/2020 for clinical indications and selected patients with an age of 19–49 years. The study design was retrospective. Our institutional review board regulations do not require approval for retrospective studies.

Inclusion criteria: (i) age 19–49 years; (ii) “high-risk” symptomatic patient: prior diagnosis of “suspected coronary artery disease” by a board-certified cardiologist within three before CTA including a physical exam, detailed record of chest pain symptoms (atypical, typical or non-specific) and their characteristics, ECG (rest and/or treadmill stress ECG) or SPECT myocardial perfusion, an echocardiography and a detailed record of the major cardiovascular risk factors (arterial hypertension, cigarette smoking, family history, dyslipidemia including total cholesterol and c-low-density lipoprotein value, diabetes and body mass index). Coronary artery disease (or coronary anomaly) was suspected based on either atypical or typical chest pain symptoms and/ or other suspicious pre-tests listed above if chest pain was absent. Suspicious findings included new onset of severe high-grade arrhythmia or prior cardiac arrest (atrial fibrillation, or others such as complex premature beats, ventricular tachycardia, new left or right bundle branch block, AV-Block ≥ II)—new onset, during treadmill and not related to other underlying known conditions. Highly positive family history of ≥1 close relative 1st order; and cardiac events or unknown sudden cardiac death at a young age (<55 years). (iii) Mild or transient troponin elevations not meeting NSTEMI or STEMI ACS criteria—without a clear presentation of myocarditis requiring differential diagnostic work-up. 

Exclusion criteria: Known coronary artery disease, including prior coronary artery bypass grafting, prior PCI/STENT, prior myocardial infarction, patients referred for congenital or structural heart disease evaluation (valves, interventional planning, cardiac devices and masses).

Coronary Computed Tomography Angiography (CTA). A non-contrast ECG-gated coronary artery calcium score (CACS) with standardized scan parameters (detector collimation 64 × 1.5 mm; 120 kV; image reconstruction 3 mm slice width, increment 1.5); prospective ECG-triggering was performed. The Agatston Score (AU) [14] was calculated. Coronary CTA was performed either with a 128-slice dual-source CTA (Definition FLASH, Siemens Healthineers, Erlangen, Germany) (2010–2020) with a detector collimation of 2 × 64 × 0.6 mm and a z-flying spot and a rotation time 0.28 s or a 64-slice CTA (Somatom 64, Siemens, Erlangen, Germany) (2005–2009) detector collimation of 64 × 0.6 mm and a rotation time of 0.33 s). Prospective ECG-triggering was used in regular heart rates < 65 bpm (70% of RR-interval) and retrospective ECG-gating in heart rates > 65 bpm and irregular rates. An iodine contrast agent (Iopromide, Ultravist 370™, Bayer Healthcare, Berlin, Germany) was injected intravenously (flow rate 4–6 mL/s + 40 cc saline), triggered into the arterial phase (bolus tracking; 100 HU threshold; ascending aorta). Contrast volume ranged from 65 to 120 cc (mean, 70–80 mL) according to the individual patient characteristics. Axial images were reconstructed with 0.75 mm slice width (increment 0.4/medium-smooth kernel B26f 3-SAFIRE) during the best diastolic and systolic phase.

Coronary CTA image analysis. Curved multiplanar reformations (cMPR) and oblique interactive MPR using client-server based 3-D post-processing software (SyngoVia^TM^, Siemens Healthineers, Erlangen, Germany) were generated:

- The coronary stenosis severity was scored qualitatively according to the CAD-RADS^TM^ score (0–5) as minimal (1) <25%, mild (2) 25–49.9%, moderate (3) 50–69.9%, severe (4) ≥70–99% and (5) occluded 100% [15] on a per-coronary segment-base (AHA-modified-17-segment classification)
- High-risk plaque (HRP) analysis: Low attenuation plaque (LAP) was defined as hypoattenuating lesion with <150 Housfield Units (HU). CT-density was screened with the “pixel lens” and the lowest HU recorded [8]. Then, a region-of-interest (ROI) of approximately 2 mm^2^ size was placed at the region of lowest density and drawn as large as possible, while sparing areas affected by artifacts or adjacent to calcifications and the CT-attenuation (HU) quantified. If a patient had multiple lesions, the one with the lowest HU was selected for a patient-based analysis. Low-attenuation plaque (LAP) was stratified into LAP < 90 HU, <60 HU (fibrofatty) [10], and LAP < 30 HU (lipid-rich necrotic core) [6,9]. The napkin-ring sign was defined as an outer high-density rim with an inner hypodense area [7]. Spotty calcification was defined as a calcification of less than 3 mm size. The remodeling index was calculated as the ratio of the maximal cross-sectional lumen of the plaque diameter and its closed proximal (or distal, e.g., in case of ostial lesions) normal reference vessel lumen diameter. Positive remodeling was defined as remodeling index > 1.1.

High-risk plaques were defined if a minimum of two out of four criteria were present (CAD-RADS/V).

**Figure 1:**
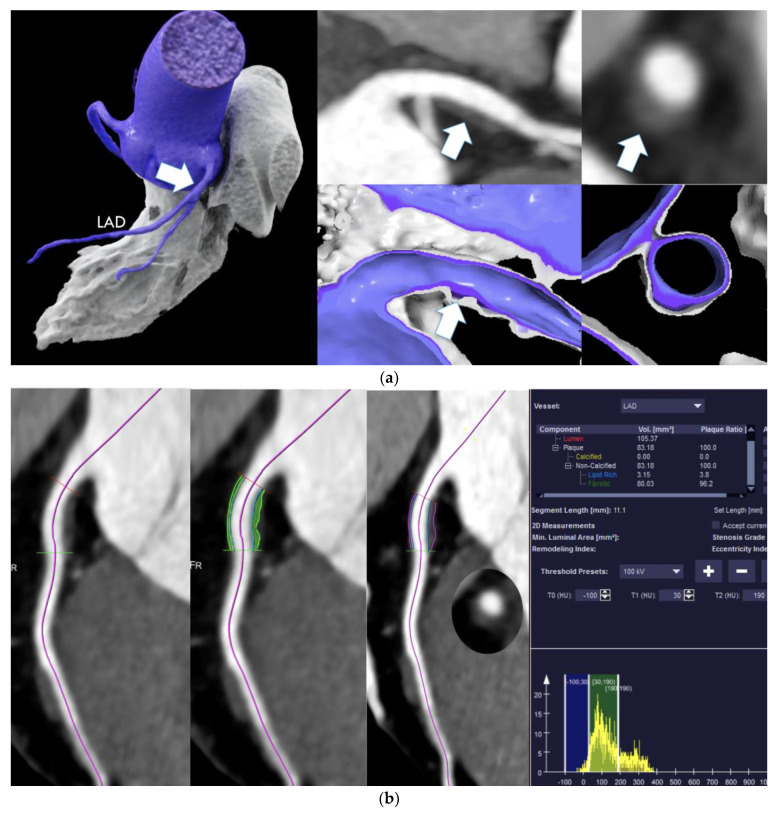
(**a**) A 30 year-old-male with diabetes since 14 years, presenting with ketoacidosis to our ED. Hs-Troponin (49 ng/dL) and HBA1c (11.2%) were elevated. CTA (A) showed a non-calcified lesion (white arrows) in the proximal LAD with positive remodeling (“high-risk- plaque”—HRP) with quantitative 3.15 mm^3^ lipid-rich necrotic core plaque volume (LAP < 30 HU) (**b**) based on CTA. CACS was 0. There was no calcific plaque components by CTA (**a**). Manual contour editing (**b**) was performed if automated tracing of lesion borders was inaccurate (mid cMPR and right cMPR) (SyngoVIA^TM^ Frontier Research, Siemens Healthineers): VRT (left) and MPR (mid and right). (**b**) cMPR and quantitative plaque analysis.

CTA analysis was performed by two independent observers (>6 months and 10 years of experience). A consensus reading was obtained. Patients with limited image quality (poor but diagnostic) due to artifacts (motion blurring, high image noise, beam hardening, or streak artifacts) were labeled as CADRADS N/6 and excluded from the following quantitative high-risk plaque analysis: All lesions scored as LAP < 60 HU were further processed by 3D post-processing research software (SyngoVIA Frontier Research, Plaque Analysis, Siemens Healthineers), and the ratio and volume of lipid-rich and fibrofatty plaques were defined (Figure 1a). Semiautomated plaque analysis was performed. The lesions were segmented fully automatically based on a coronary-tree extraction algorithm, and the plaque volume was calculated based on outer and inner wall segmentation. The accuracy of segmentation results was assured visually, and contours adjusted manually. Only lesions with a verified LAP < 30 HU component (Ratio > 1.0) based on the software were finally defined as “high-risk plaques”. 

Statistical analysis. Statistical analysis was performed using SPSS™ software (V24.0, SPSS Inc., Chicago, IL, USA). Quantitative variables are expressed as means ± standard deviation (SD), categorical variables as absolute values and percentages. Differences in all parametric data between two groups were tested using the independent *t*-test in the case of a normal distribution, or the Mann–Whitney U test was applied for non-normally distributed and rank-scaled variables; the Kruskal–Wallis test was used to test for differences in the CAD-RADS score between groups and Chi-Square for categorical data. A two-sided *p*-value less than 0.05 was considered statistically significant. 

## 3. Results

A total of 1137 patients (age, 19–49 years) were included. Table 1 shows the patient profile, CACS, and CTA results. A total of 70.5% of patients had CACS 0 and 29.5% had a positive CACS > 0.1 AU. Based on CTA, 54.4% had no atherosclerosis, 21.5% had non-obstructive coronary artery disease, and 13.7% had obstructive coronary artery disease (>50% stenosis). The rate of atherosclerosis based on CTA was significantly higher as compared to CACS (45% vs. 27%; *p* < 0.001). High-risk plaque features were found in 17.7%. Among patients with high-risk plaque, almost one-half (45.1%) had CACS 0. Among the different six age groups, there was no difference in gender (*p* = 0.407), smoking, positive family history, diabetes and body mass index. There was a small difference in dyslipidemia between age groups 1 and 2, but not between other groups. Arterial hypertension was slightly more common in patients older than 45 years. 

**Figure 2:**
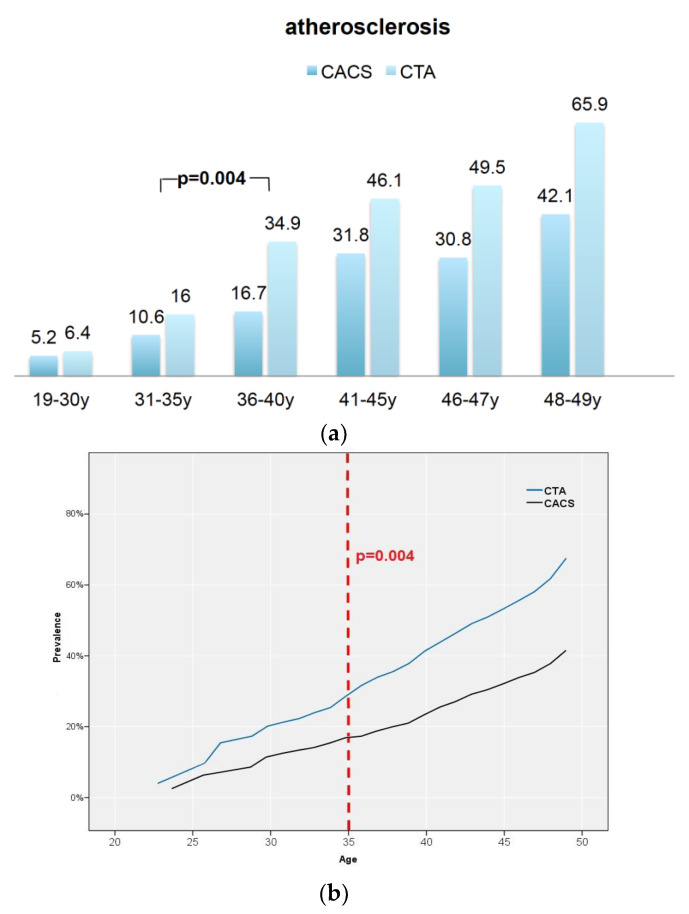
(**a**) Atherosclerosis based on the coronary artery calcium score (CACS) compared to coronary CTA. Atherosclerosis prevalence increased from 16% to 34.9% in patients older than 35 years based on CTA (*p* = 0.004) (**b**). N = percentage (%).

Table 2 shows the CACS and coronary artery disease profile based on CTA, stenosis severity, and adverse “high-risk-plaque” (HRP) features among all six age groups. Prevalence of atherosclerosis based on CTA increased significantly from 16 to 34.9% between age group 2 + 3 (31–35 y and 36–40 y) (*p* = 0.004, Chi-Square; OR 2.8: 95%CI: 1.45–5.89) and increased further between age groups 3 + 4 (*p* = 0.003; OR 0.56: 95%CI: 0.382–0.8). The rate of atherosclerosis was low and not different between the youngest age groups (19–30 y and 31–35 y) (6.4% vs. 16.0%) (Figure 2) and increased with age. 

Differences in atherosclerosis prevalence based on CACS and CTA: Below 35 years of age, the rate of atherosclerosis was low and not different between CACS and CTA (*p* = 0.703 for group 1 and *p* = 0.471 for group 2) and between group 1 and 2 (*p* = 0.209). In patients older than 35 years, the detection rate of atherosclerosis based on CTA increased compared to CACS (*p* < 0.001) for group 3 (36–40 y) and group 4 (41–45 y) (Figure 2, Table 2). 

**Figure 3:**
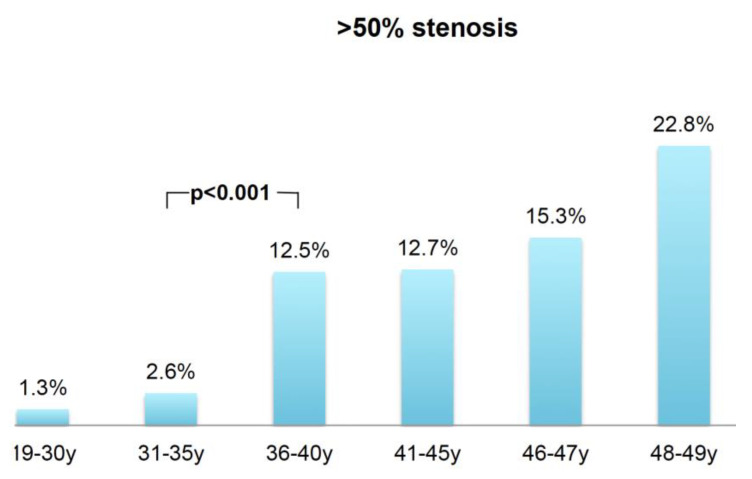
Obstructive coronary artery disease (>50% stenosis) based on CTA: Prevalence increased in patients older than 35 years of age (*p* < 0.001).

Stenosis severity (CAD-RADs^TM^): Stenosis severity was different among all six groups (*p* < 0.001). No difference (*p* = 0.220) was found between groups 1 and 2. In patients > 35 years of age, stenosis severity (CADRADS) (*p* = 0.002, Wilcoxon) increased. Between group 3 and 4 (*p* = 0.005), a further increase was observed, while no differences in CADRADS were found among age groups 1 and 2 (*p* = 0.740) and age groups 4 and 5 (*p* = 0.740). The rate of obstructive coronary artery disease was low and not different between group 1 and 2 (1.3 and 2.6%, *p* = 0.220) and increased significantly in patients older than 35 years (*p* < 0.001) (Figure 3).

**Figure 4:**
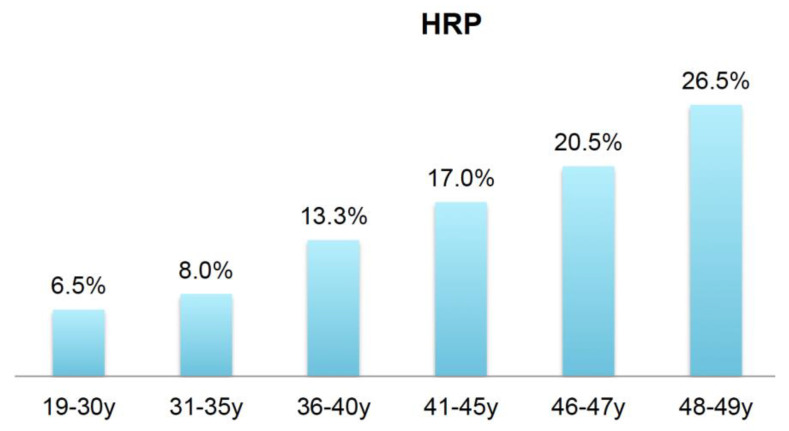
Vulnerable plaque features (high-risk-plaque—“HRP”) increased continuously with age.

High-risk plaque prevalence was low with 6.5% in the youngest age group 1 and increased linearly to 26.5% in group 6. Stepwise testing revealed no significant increase between all groups (Figure 4).

**Figure 5:**
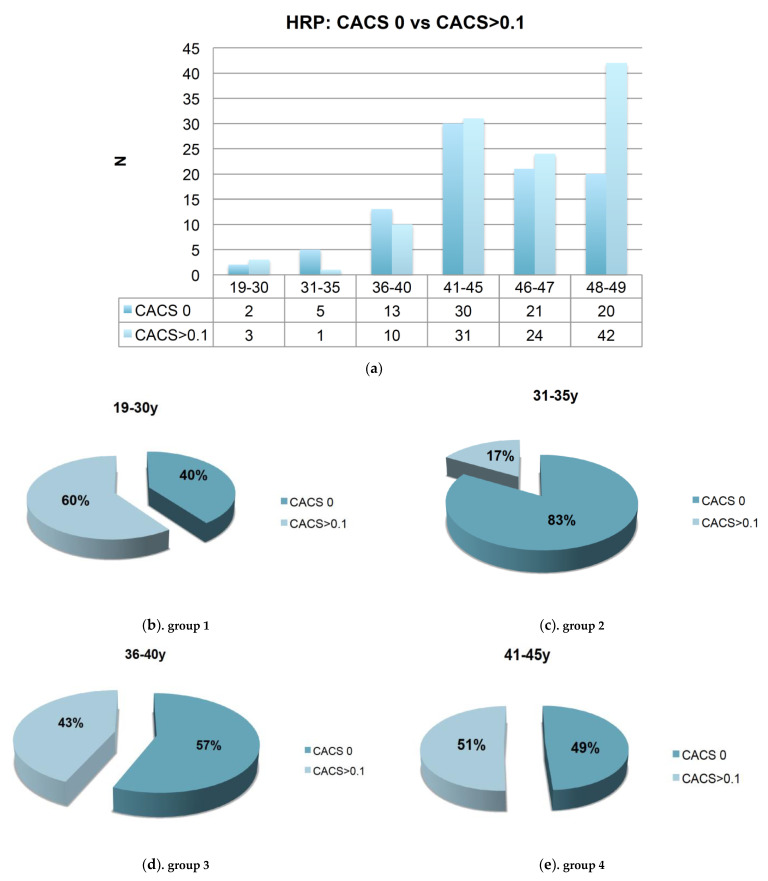
(**a**) High-risk plaque (HRP) in patients without coronary calcium (CACS 0) compared to those with coronary calcium (CACS > 0.1 AU). (**b**–**d**). The distribution of high-risk-plaque (HRP) in CACS zero compared to positive CACS > 0.1 AU among different age groups was not different with 63 vs. 36% in patients between 19 and 35 years (**b**,**c**). In patients >35 years of age, there was a trend towards an increasing prevalence of HRP in those with positive CACS > 0.1 AU (**d**,**e**).

Figure 5 shows the distribution of high-risk plaque in patients with CACS zero as compared to those with coronary calcium (CACS > 0.1 AU). The distribution was not different among age groups, with a trend towards an increasing proportion of high-risk plaque in CACS > 0.1 AU with age. Table 2 shows the coronary artery disease profile for patients with CACS 0. One quarter (24.9%) of CACS zero patients had atherosclerosis based on CTA, out of those the majority (82.5%) had nonobstructive coronary artery disease. The rate of >50% stenosis was 4.4 and 11.5% of patients with CACS 0 had high-risk plaque.

## 4. Discussion

Our observational study shows the coronary artery disease (CAD) profile based on CTA compared head-to-head with CACS in a large symptomatic young high-risk population aged 19–49 years, including “high-risk plaque = HRP” criteria. High-risk-plaques are imaging biomarkers for adverse cardiac events [5,6,7,8,9,10,16,17], independent of stenosis severity and CACS, and well-known precursor lesions for ACS [16,17].

Our data show that for patients older than 35 years, CTA clearly outperformed CACS, while in those aged between 19 and 35 years, atherosclerosis detection rates were similar and the total prevalence of CAD low. While CTA has been recently recommended as Class IA indication in the ESC guidelines of 2019 in symptomatic patients with chronic coronary syndromes and a low-intermediate-high-risk of coronary artery disease as the non-invasive modality of choice for CAD [12], the large trials published over the last decade included mainly elderly patients with a mean age of 50–60 years. Large cohort data enrolling young adults are sparse: One study enrolled asymptomatic healthy no-or-low risk Asians [13]; however, there is no study that investigated symptomatic high-risk Caucasian individuals. 

A provocative meta-analysis [18] recently proposed that CACS performs equal to CTA for the exclusion of coronary artery disease in patients with stable chest pain. However, this meta-analysis included studies in which patients underwent both CACS and CTA such as those from the CONFIRM-registry [19]. In these studies, CTA already acted as a gatekeeper to prevent adverse outcomes, by selecting those with >50% stenosis, who subsequently underwent coronary revascularization. It is therefore not clear if outcomes would have been as favorable—if the patients would have undergone CACS only. 

Beyond mortality is known to be low in adults among all age groups [4] with CACS zero, and most studies did not include milder adverse cardiac outcomes such as nonfatal myocardial infarction, ACS NSTEMI-STEMI, which occur more frequent in younger adults than death. A mild CACS of 1–10 AU was associated with a higher risk of cardiovascular death at ages < 40 years, and CACS > 10 AU was associated with a higher risk of cardiovascular and all-cause death among all age groups [4]. We found a considerable percentage of high-risk plaque features in patients with no coronary calcium, ultralow CACS 0.1–0.9, and also low CACS, with a relatively even distribution among all age groups. Adverse plaque features often cause cardiac events in both non-obstructive and obstructive coronary artery disease [17]. Our data clearly oppose a CACS-only approach to rule out coronary artery disease in a symptomatic young high-risk population, especially in those older than 35 years of age. Even in the youngest age group 19–35 y, CACS did not 100% rule out coronary artery disease. Above 35 years of age, the prevalence of atherosclerosis, obstructive disease > 50%, stenosis severity, and adverse plaque features was higher, and continuously increased stepwise until 49 years of age, pointing at increasing diagnostic yield with age.

Our cohort further allowed for a more distinct age-group based consideration of “total coronary artery disease burden” based on CTA, which is known to be associated with higher annualized event rates [20]. Between 41 and 50 years, the highest percentage of >50% stenosis was found, and in about one quarter, high-risk plaques were present. In contrast, in the age groups 19–35 years, CTA and CACS performed relatively similarly for the detection of premature atherosclerosis. The percentage of stenosis > 50% in our cohort was very low in those < 35 years (1.3 and 2.6%) but higher than that reported by Jin et al. [13] in asymptomatic healthy young Asians (0.1%) [13]. Of note, high-risk plaque was also found in the youngest age group (<35 years) but at a very low proportion (8%). High-risk plaque criteria were found in both individuals with CACS zero and positive CACS > 0.1 AU at a relatively even distribution (Figure 5). This finding highlights the superiority of CTA over CACS for cardiovascular risk stratification based, as recently shown by the SCOT HEART trial [6] and others [16,17], in summary favoring coronary CTA over CACS. Despite the strength of CACS zero [1,4], the superiority of CTA over CACS even in the absence of coronary calcium is supported by other studies [21,22,23] in adults > 50 years: In 6531 asymptomatic Asians with CACS 0 (age, mean 50 years) who underwent CTA as part of a health check-up, both fatal and non-fatal events were predicted by obstructive coronary artery disease and non-calcified plaque with lower HU, despite the low total event rate, i.e., 0.2% [21]. Similarly, in another study [22] recruiting symptomatic low-intermediate risk Caucasians (mean age 57.9 years), a higher prevalence of atherosclerosis (25.9%) was reported with a low event rate. The reason was that CTA acted as gatekeeper to select those with >50% stenosis who subsequently underwent coronary revascularization, supporting the ESC 2019 guidelines [12] rather than the meta-analysis [18]. Similar results were observed by Yu et al. [23] in an elderly symptomatic Asian population (age, 54 years, *n* = 5541) with CACS 0, in which the rate of obstructive disease was higher with 9.32% [23], than in asymptomatic individuals; however high-risk plaque were not assessed in this study. In the CONFIRM registry, CTA added incremental value over CACS for the prediction of major adverse cardiac events in CACS > 100 AU [24] only and in the elderly, but not within the lower age tertiles < 52 years. However, only stenosis severity and total plaque burden based on CTA [24] were included, and not high-risk plaque criteria, and the population was much older than ours. Importantly, our cohort represents a “high-risk” symptomatic population of young adults with suspected coronary heart disease based on pre-tests and the clinical presentation, comprising a pathological pre-test, borderline or transient Troponin elevations, a recent onset of high-grade arrhythmia or other arrhythmogenic events such as cardiopulmonary resuscitation (CPR), requiring further diagnostic workup. The minority had normal pre-tests, but in those, a very high CV-risk, such as a history of fatal premature CAD or sudden cardiac death of one or several of their close relatives <50 years of age or premature diabetes (Figure 1), was present. Osei et al. [25] identified a high number of CV risk factors (≥3) as the best predictors of a positive CAC in a small cohort of 373 young individuals (age 20–30 years), supporting our study findings. However, this study [25] did not evaluate the percentage of obstructive disease and high-risk plaque features based on CTA. Young adults will benefit from early restrictive primary prevention measures such as lifestyle modifications [26] or medical therapy. 

Study limitations. The six age groups were matched for cardiovascular risk factors, with only minor differences in arterial hypertension rates between the highest age group, and a slightly lower prevalence of dyslipidemia in the youngest < 30 years. The advantage of CACS in younger adults is a lower radiation dose. 

Therefore, in those < 30 years, the decision whether to choose CACS or CTA must be made very carefully and individually, based on the likelihood of obstructive coronary artery disease. Only very high risk and symptomatic young patients < 30 years, with a high suspicion of premature coronary atherosclerosis based on the clinical presentation (risk factors, chest pain, pre-test findings and cardiac enzyme). The radiation dose is usually still higher for CTA compared to CACS (≤1 mSv). Depending on the scanner type and patient characteristics, the radiation dose was, on average, 10 times higher for CTA when using a newer CT scanner generation, the 128-dual source CT, in our cohort. New technical developments provide the capability of performing CTA at an ultralow radiation dose exposure of 1–3 mSv, which is almost comparable to CACS. Importantly, radiation exposure is highly variable (1–15 mSv) for CTA among different CT scanner types and protocols and must be considered carefully in younger individuals. 

We did not assess the relationship between cardiovascular risk factors and the atherosclerosis profile, which has been investigated by numerous previous studies. Specific risk factors are known to promote specific plaque phenotypes [27].

## 5. Conclusions

In young symptomatic high-risk individuals older than 35 years of age, coronary CTA performed better than CACS. In those aged between 19 and 35 years, both CACS and CTA had a relatively similar detection rate of atherosclerosis, and total prevalence of atherosclerosis was low. However, CACS 0 did not reliably rule out coronary artery disease. Therefore, in very young patients, <35 years of age, individual decisions on referrals to CACS, coronary CTA or cardiac magnetic resonance imaging—depending on the specific clinical presentation (risk profile, cardiac enzymes and likelihood of ischemia)—are most reasonable.

## Figures and Tables

**Table 1:** Study population, coronary artery calcium score (CACS) and coronary CTA results.

Age (y)		42.4 ± 6.3 (19–49)
Females		346 (30.4%)
BMI (kg/m^2^)		26.1 ± 5.02
Smoking		339 (41.5%)
Arterial Hypertension		303 (37.2%)
Positive Family History		357 (44.2%)
Dyslipidemia		358 (45.1%)
Diabetes		40 (5.2%)
CACS (AU)		17.5 ± 80.3 (Range, 0–1203.5)
**CACS**		
0	70.5%	
>1.0 AU	29.5%	
**CTA-Stenosis Severity** **(CADRADS)**		
0	615 (54.4%)	
1	158 (14%)	
2	201 (17.8%)	
3	71 (6.3%)	
4 + 5	82 (7.3%)	>50% Stenosis
6/N *	4 (0.4%)	13.6%
**Atherosclerosis**		
CACS	298 (27.0%)	*p* < 0.0001
CTA	512 (45.0%)	OR: 0.44 (95%CI: 0.37–0.53)
**CTA-HRP**	202/1137 (17.7%)	
CACS 0	91 (45.1%)	
CACS 0.1–0.9	31 (15.3%)	
CACS > 1.0	80 (39.6%)	
**CACS 0** **N = 802**		
**CAD by CTA**		
CADRADS 0	200 (24.9%)	
1	600 (74.8%)	
2	87	
3	78	
4 + 5	17	
6/N *	18	>50% Stenosis
HRP	2 (0.3%)	35/802
	92 (11.5%)	(4.4%)

Abbreviations. CAD = coronary artery disease. CACS = coronary artery calcium score. HRP = high-risk plaque. CADRADS = coronary artery stenosis severity score [15]. AU = Agatston Score. N = nondiagnostic. CTA = coronary computed tomography angiography. 6/N * = nondiagnostic image quality within 1–5 coronary segments not involving the main/proximal. Ordinal data are shown as N = count (%). Parametric data are displayed as mean ± SD. BMI = body mass index.

**Table 2:** Atherosclerosis in 6 age groups: coronary artery calcium score (ACS) and coronary CTA results.

	19–30 y Group 1 *n* = 77	31–35 y Group 2 *n* = 75	36–40 y Group 3 *n* = 173	41–45 y Group 4 *n* = 358	46–47 y Group 5 *n* = 220	47–48 y Group 6 *n* = 234
**CACS**						
0	92.2%	88.2%	86.2%	68.2%	69.2%	57.9%
>=0.1 AU	5.2%	10.6%	13.8%	31.8%	30.8%	42.1%
Cat 1 0.1–10	4 (5.2%)	7 (9.3%)	17 (9.8%)	53 (14.8%)	34 (15.5%)	37 (15.8%)
2 10–100	0 (0%)	0 (0%)	3 (1.7%)	29 (8.1%)	22 (10%)	31 (13.2%)
3 100–300	0 (0%)	1 (1.4%)	3 (1.7%)	15 (4.2%)	6 (2.7%)	21 (9.1%)
4 >300	0 (0%)	0 (0%)	1 (0.58%)	3 (8.4%)	3 (1.4%)	7 (2.9%)
**CACS (AU)** (range)	0.03 ± 0.02 (0–1.5)	1.42 ± 11.8 (0–102)	11.5 ± 89.1 (0–1101.4)	15.4 ± 54.1 (0–420)	18.1 ± 82.0 (0–758.1)	35.7 ± 11.9 (0–1203.5)
**CAD-RADS**						
0	70 (90.9%)	63 (84%)	112 (65.1%)	181 (50.7%)	110 (50%)	80 (34.1%)
1	3 (3.9%)	5 (6.7%)	20 (11.6%)	58 (16.2%)	29 (13.2%)	42 (17.9%)
2	1 (1.3%)	5 (6.7%)	18 (10.5%)	70 (19.6%)	49 (22.3%)	61 (26%)
3	0%	1 (1.3%)	11 (6.4%)	17 (4.8%)	14 (6.4%)	29 (12.4%)
4 + 5	1 (1.3%)	1 (1.3%)	10 (5.8%)	31 (8.7%)	18 (8.3%)	22 (9.4%)
6/N	2 (2.6%)	63 (84%)	1 (0.6%)	181 (50.7%)	110 (50%)	80 (34.1%)
**Atherosclerosis-CTA**	6.4%	16.0%	34.9%	46.1%	49.5%	65.9%
**HRP**	5 (6.5%)	6 (8%)	23 (13.3%)	61 (17.0%)	45 (20.5%)	62 (26.5%)
CACS 0	2	5	13	30	21	20
CACS 0.1–0.9 AU	3	1	3	12	4	8
CACS > 1.0 AU	0	0	7	19	20	34

Abbreviations: CACS = coronary artery calcium score. AU = Agatston Units. HRP = high–risk plaque. CTA = computed tomography angiography. 6/N = partial nondiagnostic image quality (in 1 up to 5 coronary segments, not involving the main/proximal).

## Data Availability

The data have not been made publicly available. Data are stored locally and can be provided upon request.

## Abbreviations

Coronary computed tomography angiography (CTA), coronary artery calcium score (CACS), Agatston Units (AU), Hounsfield Units (HU), high-risk plaque (HRP), low-attenuation plaque (LAP), European Society of Cardiology (ESC).
